# Resistibility

**Published:** 1896-02

**Authors:** G. A. Mills

**Affiliations:** New York


### "RESISTIBILITY."

BY DR. G. A. MILLS, NEW YORK.

We are indebted to the late Professor Garretson for the use of this term, and we think that a consideration of its wide-spread application might be profitable. We are sure that as dentists we are widely at fault in the care of our patient's humane interests. Doubtless the cause of this lies in the fact that mechanics have ruled a larger part of our procedure in practice. The need of the hour was never so apparent that we should know what to do and why we should do it.

True science has not been our practical leader. While a few have had the ambitious industry to investigate, too many more have laid back and cavilled at the impracticability of "so much science." But who will have the hardihood to say that these delving labors have not caused us to be face to face with the stern necessity of true scientific knowledge, so that the best may be done with our daily practice? We should say thanks to those who have given us some facts that now form a nucleus for future, better doings. Dealing with life is a more serious and responsible affair than many take into their thoughtful consideration,—some from a lack of thought, and many more because of their mad race for gain. We wish that the hope for better things might be looked for among the mass as the advance along the lines of a more liberal culture that just now we hear emphasized is carried out. "Resistibility," homely interpreted, means how much can the bodies bear from us in the application of what we apply as remedial. Disease always advances in the lines of least resistance. Some bodies are so fortified that a vast amount of malpractice will make no inroad, while others are easily affected and laid under great stress of danger, sometimes beyond the possibility of recovery. We are constantly coming in contact with such evidences. "Health is the most perfect germicide." This maxim embodies the whole subject in a nutshell. Idiosyncrasies are more or less exceptions to the rule that is considered general. The application of this knowledge, found in the term we are considering, is invaluable in making a diagnosis. We say that some are born with endowments,—special, of course.

They have an advantage; but study and observation will, or can, enhance an acquired ability. Some have not the capacity ever to attain the ability for helpful service to living tissues. They have no surgical capacity. As an illustration: In a case where alveolar abscess had been dealt with successfully, but the practitioner did not know it, and had vigorously used the bur, and the result was that he had produced a large ridge of scar tissue which he mistook for a further disturbance. The patient died not long after, not because of this blunder, yet had he lived he would have gone on inflicting his ignorance. Never was there more need of care and caution in the thought of our subject than these days of parlor machinery that is so manifested in practice. We do believe in the value of motor power in our practice, but we also tremendously believe it is shockingly overdone. Too much of this is for effect, and some in order to be on a footing of our neighbors. It should be always our desire to perform operations within the line of the least infringement of sacrifice in time and endurance that may be essential for the best results. Indefinitely we could enumerate the lines of application of this term.

This word was one of the outcomes of the late meeting of the New Jersey State Society at Asbury Park, and we have felt like italicizing it every time it has come into our thought. We only trust some one will emphasize it more effectively than we have.

